# Action-Specific Disruption of Perceptual Confidence

**DOI:** 10.1177/0956797614557697

**Published:** 2015-01

**Authors:** Stephen M. Fleming, Brian Maniscalco, Yoshiaki Ko, Namema Amendi, Tony Ro, Hakwan Lau

**Affiliations:** 1 Center for Neural Science, New York University; 2 Department of Experimental Psychology, University of Oxford; 3 Department of Psychology, Columbia University; 4 National Institute of Neurological Disorders and Stroke, National Institutes of Health, Bethesda, Maryland; 5 Department of Psychology, The City College of the City University of New York; 6 Department of Psychology, University of California, Los Angeles

**Keywords:** perception, motor processes, monitoring, open data

## Abstract

Theoretical models of perception assume that confidence is related to the quality or strength of sensory processing. Counter to this intuitive view, we showed in the present research that the motor system also contributes to judgments of perceptual confidence. In two experiments, we used transcranial magnetic stimulation (TMS) to manipulate response-specific representations in the premotor cortex, selectively disrupting postresponse confidence in visual discrimination judgments. Specifically, stimulation of the motor representation associated with the unchosen response reduced confidence in correct responses, thereby reducing metacognitive capacity without changing visual discrimination performance. Effects of TMS on confidence were observed when stimulation was applied both before and after the response occurred, which suggests that confidence depends on late-stage metacognitive processes. These results place constraints on models of perceptual confidence and metacognition by revealing that action-specific information in the premotor cortex contributes to perceptual confidence.

A dominant view is that perceptual confidence is determined by the strength of incoming sensory signals (Barthelme & Mamassian, 2010; Kepecs, Uchida, Zariwala, & Mainen, 2008; Kiani & Shadlen, 2009; Vickers, 1979; Zylberberg, Barttfeld, & Sigman, 2012; see Yeung & Summerfield, 2012, for a review). However, perceptual-decision signals are also seen in neural circuits specialized for motor actions (Cisek & Kalaska, 2005; Freedman & Assad, 2011; Hernandez, Zainos, & Romo, 2002; Romo, Hernandez, & Zainos, 2004; Shadlen & Newsome, 2001), which suggests that the motor system may also contribute to visual confidence. Indeed, in dominant models of perceptual decision making (Gold & Shadlen, 2007), sensory evidence is accumulated in “embodied” effector-specific circuits, and confidence increases in proportion to the amount of evidence supporting one response over another (Kiani & Shadlen, 2009; Vickers, 1979). This view raises the novel and counterintuitive prediction that directly interfering with action-specific representations may alter confidence in a visual discrimination. Moreover, as metacognitive accuracy depends on the trial-by-trial correspondence between confidence and performance, such action-specific interference may also impair metacognitive accuracy. The demonstration of such effects would shed light on the mechanisms of confidence and metacognition.

In the present research, we set out to test this hypothesis. We applied unilateral single-pulse transcranial magnetic stimulation (TMS) to the dorsal premotor cortex (PMd) of participants during a difficult perceptual discrimination. Participants were asked to report, using their left or right hand, their visual discrimination response (which side of the screen contained a grating stimulus superimposed on noise in Experiment 1; whether a central grating was horizontally or vertically oriented in Experiment 2) and provide a confidence rating for their discrimination. On each trial, a single TMS pulse was delivered at one of two time points—either 100 ms after stimulus onset or immediately following the response (Fig. 1). As the premotor cortex is known to contain lateralized motor representations (Passingham, 1993) and is directly connected to the lateral prefrontal cortex, which has been linked to metacognition (Fleming, Huijgen, & Dolan, 2012; Fleming, Weil, Nagy, Dolan, & Rees, 2010; Rounis, Maniscalco, Rothwell, Passingham, & Lau, 2010), we hypothesized that TMS would affect visual confidence (and therefore metacognitive accuracy) differently depending on whether the hand participants responded with was contralateral to (congruent trials) or ipsilateral to (incongruent trials) the hemisphere that was being stimulated.

**Fig. 1.**
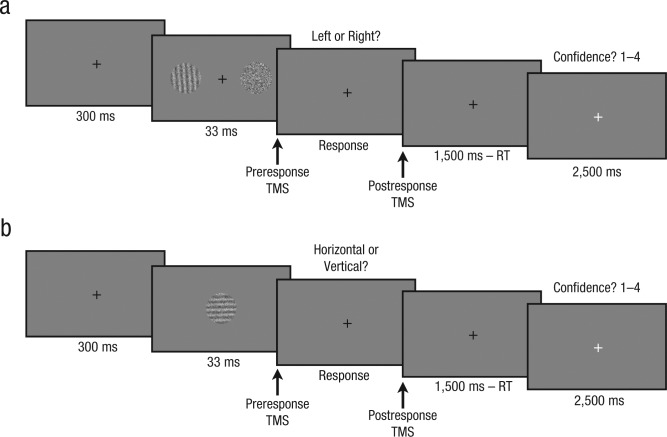
Example trial sequence from (a) Experiment 1 and (b) Experiment 2. In Experiment 1, participants briefly viewed two noise patches, on one of which a grating was superimposed, and subsequently had to indicate the side on which the grating was presented. In Experiment 2, participants briefly viewed a centrally presented grating and then had to indicate whether it had been horizontally or vertically oriented. A single pulse of transcranial magnetic stimulation (TMS) was applied either 100 ms after stimulus onset (preresponse condition) or immediately after participants’ response (postresponse condition). The hemisphere that received TMS was counterbalanced across participants. Following each response, participants were asked to provide a confidence rating on a scale from 1 (*low confidence*) to 4 (*high confidence*) 1,500 ms after the offset of the stimulus. RT = response time.

The inclusion of pre- and postresponse-stimulation conditions allowed us to assess whether metacognitive confidence depends on continued processing of effector-specific evidence after the decision has been made. In decisional-locus models, confidence is based on the evidence available at the time of the judgment (Kiani & Shadlen, 2009; Vickers, 1979). In postdecisional-locus models, confidence also depends on the accumulation of evidence after the decision (Pleskac & Busemeyer, 2010). A demonstration that response-specific TMS affects confidence even when applied after the decision would support postdecisional-locus accounts of confidence.

We found that, in two experiments, both pre- and postresponse TMS to PMd affected visual confidence in a response-dependent manner, with incongruence between TMS and participants’ response reducing confidence and metacognitive accuracy. In an otherwise identical experiment, applying TMS to primary motor cortex (M1) did not affect confidence. Together, our results reveal that action-specific information in the premotor cortex contributes to perceptual confidence.

## Experiment 1

### Method

#### Participants

Participants were healthy volunteers with normal or corrected-to-normal vision and no history of neurological or psychiatric disorders. We based our sample size on a previously published TMS study from our laboratory (Rounis et al., 2010). We decided prior to data collection to test at least 20 participants in each experiment. Twenty-five participants completed the PMd experiment, and 23 completed the M1 experiment. We excluded any participant with more than 20% missed responses overall (*n* = 12). An additional 2 participants were excluded because of unstable threshold estimation leading to ceiling performance (> 90%) in the main task. Thus, 17 participants contributed data to the PMd group (10 females, 7 males; mean age = 23.5 years, *SD* = 7.5), and 17 participants contributed data to the M1 group (10 females, 7 males; mean age = 23.5 years, *SD* = 2.8). In the final sample, 11 participants in each group received right-hemisphere stimulation. The experiment was approved by the Columbia University Institutional Review Board.

#### Equipment

Participants were seated 60 cm in front of an iMac monitor (24-in. LCD, 1,920-pixel × 1,200-pixel resolution, 60-Hz refresh rate) and responded using the standard Apple keyboard. Stimuli were generated using the Psychophysics Toolbox (Brainard, 1997; Pelli, 1997) in MATLAB (The MathWorks, Natick, MA). A standard figure-of-eight coil with 70-mm circular components (MagStim, Whitland, United Kingdom) was used to deliver single-pulse TMS.

#### Stimuli and procedure

Experiment 1 was carried out at the Department of Psychology, Columbia University. In the main experiment, participants received TMS over PMd. We also carried out a separate control experiment in which the stimulation site was moved to M1. The procedure for both experiments was otherwise identical. For both groups, TMS was applied unilaterally, with stimulation side counterbalanced between participants.

On each trial, two stimuli were presented 4° on either side of fixation for 33 ms (Fig. 1). Each stimulus was a circle (3° diameter) containing randomly generated white noise. The target stimulus was a randomly oriented sinusoidal grating (2 cycles per degree) embedded in one of the noise patches. After the offset of the stimuli, participants provided a forced-choice judgment as to whether the left or right stimulus contained the target grating by pressing, respectively, the “f” key with their left hand or the “j” key with their right hand. On half the trials, participants received a single TMS pulse 100 ms after stimulus onset (preresponse condition), whereas on the other half, TMS was applied immediately after participants made their response (postresponse condition). These conditions were randomly interleaved. Following the 1,500-ms response period, participants were asked to provide a confidence rating on a scale from 1 (*low confidence*) to 4 (*high confidence*) by pressing the “j,” “k,” “l,” or “;” keys, respectively, using their right hand. Participants were encouraged to use the full range of the confidence scale. Participants were instructed to maintain fixation on a central crosshair (subtending 0.35°) for the duration of the trial.

The experiment consisted of 400 trials split into four blocks of 100 trials each. Rest periods were interleaved between blocks. Prior to the start of the main experimental session, grating contrast was adjusted to yield threshold performance for each individual participant using the QUEST procedure (Watson & Pelli, 1983). The contrast of the target stimulus was defined as the sum of a grating with Michelson contrast C_grating_ and a patch of visual noise with Michelson contrast C_noise_. The total contrast of the target stimulus, C_target_ = C_grating_ + C_noise_, was set to 0.9. The nontarget stimulus was also set to a Michelson contrast of 0.9. The QUEST procedure estimated the ratio of the grating contrast to the noise contrast (R_grating_ = C_grating_/C_noise_) that yielded 70% correct performance. Three independent threshold estimates of R_grating_ were acquired, with 40 randomly ordered trials contributing to each. Confidence ratings were also collected while threshold performance was determined, but they were not analyzed further. The median value of these threshold estimates was used to set the value of R_grating_ in the main experiment, which remained constant.

#### TMS protocol

To prepare participants for TMS, we first marked the intersection of the nasion-inion line and the interaural line with ink on a swim cap as Point X, in line with the 10-20 system (Meyer, Britton, Kloten, & Steinmetz, 1991). A mark was also made 5 cm to the left or right of Point X on the interaural line (Point A; Mills & Nithi, 1997). The position on the motor cortex that elicited a finger or arm twitch with the minimal stimulus intensity was then located. We initially set the intensity to 70% of the maximum stimulator output, decreasing it by 5% while moving the coil anteroposteriorly and mediolaterally in relation to Point A in steps of 0.5 cm. With each decrease, a single pulse was delivered. Decreases and movements were made until the lowest percentage intensity at which at least 5 out of 10 stimulations resulted in an observable twitch of the finger, arm, or wrist was found. This position (the *motor hot spot*) was labeled (Point B), and the minimal intensity was recorded as the active motor threshold (AMT).

The site of PMd stimulation was 3 cm anterior to Point B on a line parallel to the midsaggital line. Previous studies have shown good correspondence between scalp positions 2 to 3 cm anterior to the motor hot spot and the underlying dorsal premotor complex (Bestmann, Baudewig, Siebner, Rothwell, & Frahm, 2005; Chen, Mima, Siebner, Oga, & Hara, 2003; Johansen-Berg et al., 2002; O’Shea, Sebastian, Boorman, Johansen-Berg, & Rushworth, 2007; Schluter, Rushworth, Passingham, & Mills, 1998). M1 stimulation was located at Point B. Single pulses were delivered at a stimulation intensity of 90% of AMT.

#### Data analysis

No statistical analyses were conducted prior to the completion of testing for all participants. Participants’ responses were classified according to whether they were contralateral (congruent trials) or ipsilateral (incongruent trials) to the TMS pulse and whether they were correct or erroneous. Response times (RTs) were measured to the stimulus, as well as between the onset of the confidence-rating screen and the confidence rating. Effects of condition on confidence ratings and RTs were assessed via hierarchical linear mixed-effects regression using the lme4 package in R (Version 3.0.1; Bates, Maechler, Bolker, & Walker, 2014). We obtained *p* values for regression coefficients using the *car* package (Fox & Weisberg, 2011). Mixed-effects logistic regression was used to estimate effects of condition on response accuracy. All effects were taken as random at the participant level, and condition estimates and statistics reported are at the population level. Predictors were coded as follows—accuracy: error = 0, correct = 1; congruence: incongruent = 0, congruent = 1; time: preresponse = 0, postresponse = 1. We calculated 95% confidence intervals (CIs) on summary statistics using a bootstrap procedure (Efron & Tibshirani, 1993).

### Results and discussion

In our analyses, the congruence between the TMS pulse and participants’ response (congruent, incongruent) was crossed with the time when TMS was applied (before the response, after the response). Participants used the full range of the 4-point confidence scale (Table S3 in the Supplemental Material available online). We carried out a factorial analysis of both discrimination performance and subjective confidence.

**Fig. 2.**
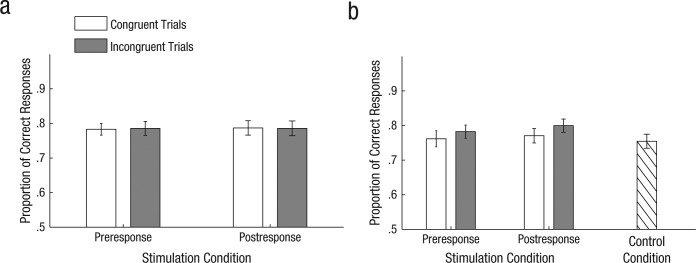
Mean proportion of correct responses in the group that received transcranial magnetic stimulation (TMS) in the dorsal premotor cortex in (a) Experiment 1 and (b) Experiment 2. Results are shown separately for congruent and incongruent trials when TMS was delivered before or after participants’ response. In (b), results are also shown for a control condition (in which TMS was not applied). Error bars reflect standard errors of the mean.

For the PMd group, neither TMS-response congruence nor time of stimulation influenced visual discrimination performance (Fig. 2a; congruence: β = −0.02, *SE* = 0.17, *p* = .91; time: β = 0.0002, *SE* = 0.09, *p* = .99). In addition, despite the TMS pulse being lateralized, it did not induce overt response bias (95% CI for percentage of contralateral responses: preresponse condition = [43%, 55%]; postresponse condition = [44%, 56%]).

**Fig. 3.**
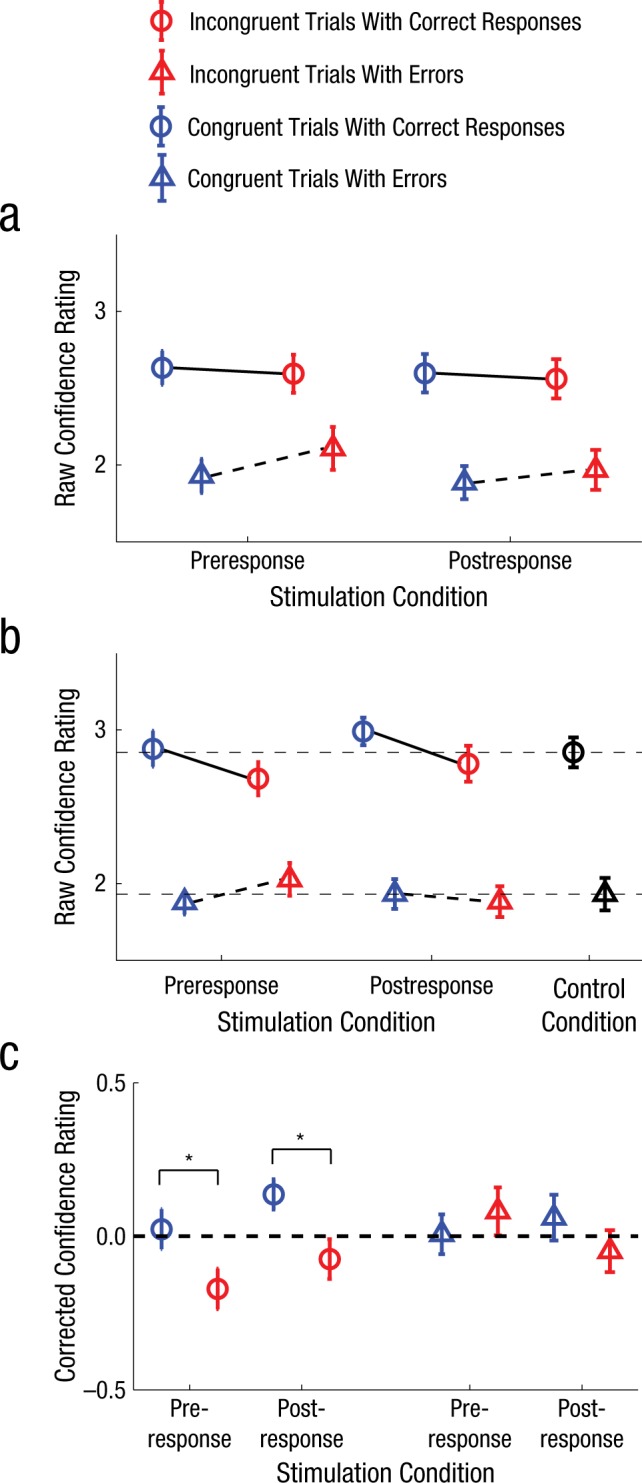
Confidence ratings in the group that received transcranial magnetic stimulation (TMS) in the dorsal premotor cortex in (a) Experiment 1 and (b, c) Experiment 2. The graphs in (a) and (b) show raw mean confidence ratings as a function of stimulation condition, response accuracy, and congruence. In (b), results are also shown for a control condition (in which TMS was not applied) for trials responded to correctly and incorrectly. Dashed lines reflect mean confidence in the control condition. The graph in (c) shows baseline-corrected confidence data in Experiment 2 as a function of response accuracy. Baseline-corrected confidence data were calculated by subtracting mean raw confidence ratings on no-TMS trials from mean raw confidence ratings on TMS trials. Error bars reflect standard errors of the mean. Asterisks indicate a significant difference between TMS conditions (*p* < .05).

To understand how TMS affected trial-by-trial subjective confidence, we constructed a mixed-effects regression model to explain confidence using binary predictors for congruence, time, and accuracy (Table 1). As expected, we found a significant main effect of accuracy on confidence, with greater confidence on trials with correct responses than on trials with errors (*p* < .001). Crucially, we also found significant effects of congruence: Participants reported lower confidence overall when TMS was incongruent with their response than when it was congruent (*p* < .05). In addition, there was a significant interaction of congruence and accuracy: Confidence was lower on incongruent trials with correct responses but higher on incongruent trials with errors (*p* < .05; Fig. 3a; see also Table S1 in the Supplemental Material). No main effects or interactions with time were found (*p* > .29), which indicates that the action-specific effects of TMS on confidence are not specific to its delivery before or after a perceptual decision.

**Table 1.** Results From Experiment 1: Regression Analyses Predicting Confidence From Accuracy, Congruence, and Time in the Dorsal-Premotor-Cortex (PMd) Group

Predictor	*b*	*p*
Intercept	2.08 (0.14)	< .0001**
Accuracy	0.52 (0.10)	< .0001**
Congruence	−0.19 (0.08)	.03*
Time	−0.08 (0.08)	.29
Accuracy × Congruence	0.22 (0.09)	.01*
Accuracy × Time	0.04 (0.09)	.66
Congruence × Time	0.08 (0.11)	.45
Accuracy × Congruence × Time	−0.07 (0.12)	.54

Note: Standard errors are given in parentheses. Predictors were coded as follows—accuracy: error = 0, correct = 1; congruence: incongruent = 0, congruent = 1; time: preresponse = 0, postresponse = 1.

* *p* < .05. **p < .01.

Congruence did not affect measures of RT to either the stimulus (β = −0.008, *SE* = 0.03, *p* = .78) or the confidence rating (β = 0.03, *SE* = 0.03, *p* = .33), which makes it unlikely that effects on metacognition were mediated by effects of TMS on details of the action itself (see Fig. S1 and Tables S4 and S5 in the Supplemental Material); there were main effects of stimulation time on RT resulting from a general speeding effect of preresponse TMS—decision RT: β = 0.07, *SE* = 0.02, *p* < .01; rating RT: β = 0.08, *SE* = 0.02, *p* < .001. No effects of M1 TMS congruence on confidence were observed (Table 2; all *p*s > .16), despite a very similar pattern of TMS effects on RTs (Fig. S2 in the Supplemental Material). In a regression model including stimulation location as a between-groups factor, the critical three-way interaction among location, congruency, and accuracy was significant (β = 0.19, *SE* = 0.09, *p* < .05), which indicates that action-specific effects of TMS on confidence were observed at PMd but not M1.

**Table 2.** Results From Experiment 1: Regression Analyses Predicting Confidence From Accuracy, Congruence, and Time in the Primary-Motor-Cortex (M1) Group

Predictor	*b*	*p*
Intercept	1.80 (0.11)	< .0001**
Accuracy	0.46 (0.07)	< .0001**
Congruence	−0.08 (0.09)	.35
Time	0.006 (0.07)	.93
Accuracy × Congruence	−0.03 (0.10)	.80
Accuracy × Time	−0.01 (0.08)	.88
Congruence × Time	−0.06 (0.10)	.56
Accuracy × Congruence × Time	0.05 (0.13)	.69

Note: Standard errors are given in parentheses. Predictors were coded as follows—accuracy: error = 0, correct = 1; congruence: incongruent = 0, congruent = 1; time: preresponse = 0, postresponse = 1.

** *p* < .01.

Together, our results demonstrate that interfering with premotor representations associated with responses in a visual discrimination task leads to systematic changes in confidence despite discrimination accuracy remaining unchanged. One potential mechanism mediating these effects is that TMS interferes with sensory evidence in embodied, effector-specific circuits both before and after a choice has been made. However, we also considered an alternative explanation. The design of Experiment 1 required participants to decide whether the left or right visual hemifield contained a grating patch. As PMd is adjacent to the frontal eye fields, which contain representations of contralateral visual space (Mohler, Goldberg, & Wurtz, 1973), it is possible that lateralized TMS pulses led to visual attentional biases that affected confidence to a greater degree than decision accuracy. In Experiment 2, we examined the effect of premotor TMS on a nonspatial version of the task to rule out this nonmotoric explanation.

## Experiment 2

Experiment 2 was similar to Experiment 1, with two alterations. First, the task required a nonspatial judgment as to whether a centrally presented grating was oriented horizontally or vertically. Second, we included a baseline condition without TMS on one-fifth of trials.

### Method

#### Participants

Data were collected from 20 healthy volunteers with normal or corrected-to-normal vision and no history of neurological or psychiatric disorders. The same exclusion criteria were applied as in Experiment 1: 2 participants were excluded due to unstable threshold estimation leading to ceiling performance in the main task. Eighteen participants’ data were analyzed (8 females, 10 males; mean age = 25.4 years, *SD* = 4.4). In the final sample, 8 participants received right-hemisphere stimulation. The study was approved by the City University of New York Institutional Review Board.

#### Equipment

The stimuli were presented at a distance of 57 cm on a Sony Triniton 17-in. CRT monitor with a refresh rate of 60 Hz. As in Experiment 1, the Psychophysics Toolbox and MATLAB were used to generate the stimuli, and a standard figure-of-eight coil with 70-mm circular components was used to deliver single-pulse TMS.

#### Stimuli and procedure

Experiment 2 was carried out at the Department of Psychology at The City College of the City University of New York. A single low-contrast grating was presented centrally on each trial (Fig. 1b). Participants made a forced-choice judgment as to whether the grating was oriented horizontally or vertically by pressing, respectively, the “f” key with their left hand or the “j” key with their right hand on a standard keyboard. As in Experiment 1, a single TMS pulse was delivered at one of two time points—100 ms after stimulus onset (preresponse condition) or immediately following participants’ response (postresponse condition). TMS was applied unilaterally, and only over PMd, with stimulation side counterbalanced between participants. Following their response, participants were asked to provide a confidence rating on a scale from 1 (*low confidence*) to 4 (*high confidence*) by pressing the “h,” “j,” “k,” or “l” keys, respectively, using their right hand. Prior to the experiment, the contrast of the grating was titrated using the QUEST procedure, as described for Experiment 1.

The main experiment consisted of 500 trials split into five blocks of 100 trials each. Two hundred preresponse-TMS trials, 200 postresponse-TMS trials, and 100 no-TMS trials were randomly interleaved. The TMS protocol was identical to that in Experiment 1.

### Results

As in Experiment 1, neither congruence nor time of stimulation influenced visual discrimination performance (Fig. 2b; congruence: β = −0.13, *SE* = 0.13, *p* = .34; time: β = 0.12, *SE* = 0.09, *p* = .18), and there was no evidence that TMS pulses induced a contralateral bias in responding (95% CI for percentage of contralateral responses—preresponse condition = [47%, 55%]; postresponse condition = [49%, 56%]). Effects of TMS on RTs to both the decision and confidence rating were similar to those observed in Experiment 1 (Fig. S1).

Crucially, despite the task requiring a nonspatial judgment, in Experiment 2, we replicated the effects of TMS-response congruence on confidence observed in Experiment 1. Specifically, we found a significant interaction of congruence and accuracy (*p* < .05; Fig. 3b). Again as in Experiment 1, we observed no interactions of TMS with time. The main effect of congruence did not reach significance in Experiment 2 (*p* = .09; see Table 3).

**Table 3.** Results From Experiment 2: Regression Analyses Predicting Confidence From Accuracy, Congruence, and Time in the Dorsal-Premotor-Cortex (PMd) Group

Predictor	*b*	*p*
Intercept	2.06 (0.11)	< .0001**
Accuracy	0.63 (0.11)	< .0001**
Congruence	−0.13 (0.08)	.09
Time	−0.13 (0.08)	.12
Accuracy × Congruence	0.32 (0.13)	.01*
Accuracy × Time	0.22 (0.10)	.02*
Congruence × Time	0.18 (0.11)	.10
Accuracy × Congruence × Time	−0.16 (0.14)	.23

Note: Standard errors are given in parentheses. Predictors were coded as follows—accuracy: error = 0, correct = 1; congruence: incongruent = 0, congruent = 1; time: preresponse = 0, postresponse = 1.

* *p* < .05. **p < .01.

The addition of a no-TMS control condition in Experiment 2 allowed us to quantify changes in confidence on congruent and incongruent trials relative to a neutral baseline. We baseline-corrected the confidence data on TMS trials by subtracting out the mean confidence on no-TMS trials (shown in Fig. 3b) for each individual participant. From this analysis, we found that TMS congruency exerted predominant effects on trials with correct responses rather than on trials with errors (Fig. 3c).

## Metacognitive Efficiency

The effect of TMS on confidence in both Experiments 1 and 2 suggests that PMd stimulation may have consequences for metacognitive efficiency or the ability to discriminate between one’s own correct and incorrect responses. We computed a model-based measure of metacognitive efficiency, meta-*d*′/*d*′ (Maniscalco & Lau, 2012). This approach takes into account changes in basic task performance (*d*′) that may influence metacognitive performance (meta-*d*′; Galvin, Podd, Drga, & Whitmore, 2003; Maniscalco & Lau, 2012), thereby obscuring to what extent changes in meta-*d*′ originate from changes in the functioning of metacognitive mechanisms per se, rather than being driven by the quality of the perceptual input into such mechanisms. Meta-*d*′/*d*′ controls for such potential confounds by measuring participants’ metacognitive performance (meta-*d*′), given their performance on the perceptual task (*d*′). When meta-*d*′/*d*′ = 1, participants can be said to be metacognitively optimal.

Pooling data from both PMd experiments, we estimated meta-*d*′ separately for each TMS condition (see the Supplemental Material for full details of this procedure). Effects of condition on meta-*d*′/*d*′ were assessed with mixed-effects analyses of variance (ANOVAs) in R with the factors experiment (1, 2), TMS-response congruency (congruent, incongruent), and stimulation time (preresponse, postresponse).

**Fig. 4.**
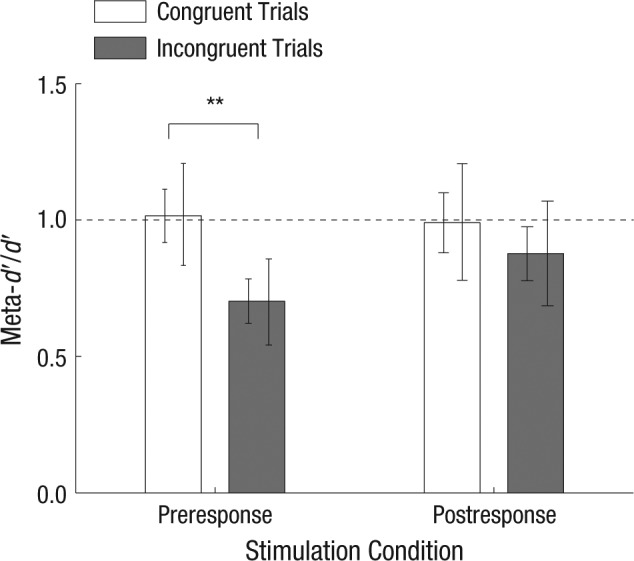
Metacognitive efficiency (meta-*d*′/*d*′) as a function of stimulation condition and congruence in the group that received transcranial magnetic stimulation (TMS) in the dorsal premotor cortex, pooled across Experiments 1 and 2. For each data bar, error bars on the left reflect standard errors of the mean, and error bars on the right reflect bootstrapped 95% confidence intervals. The dashed line indicates the optimal meta-*d*′/*d*′ value. Asterisks indicate a significant difference between conditions (*p* < .01).

Compared with congruent TMS, incongruent TMS was found to significantly reduce meta-*d*′/*d*′, *F*(1, 33) = 5.91, *p* = .02, η_*p*_^2^ = .15 (Fig. 4; see also Table S2 in the Supplemental Material). This effect held when we analyzed meta-*d*′ uncorrected by *d*′, *F*(1, 33) = 4.60, *p* = .04, η_*p*_^2^ = .12. Examining paired comparisons between the pre- and postresponse conditions, only preresponse TMS was found to interfere with metacognitive efficiency, preresponse: *t*(34) = 3.12, *p* = .004; postresponse: *t*(34) = 0.95, *p* = .35, and when compared with an optimal meta-*d*′/*d*′ value of 1, only incongruent TMS delivered before the response led to suboptimal metacognitive efficiency (95% CI = [0.54, 0.86]). However, the absence of a postresponse effect of TMS on meta-*d*′/*d*′ should be interpreted with caution, as the Congruence × Time interaction was not significant, *F*(1, 34) = 2.21, *p* = .15, η_*p*_^2^ = .06. The effect of TMS congruence on meta-*d*′/*d*′ was consistent across experiments—interaction with experiment: *F*(1, 33) = 0.70, *p* = .41, η_*p*_^2^ = .02 (Fig. S3 in the Supplemental Material). In keeping with an absence of an effect of M1 TMS on confidence, results showed that M1 stimulation had no effect on metacognitive efficiency, *F*(1, 16) = 0.13, *p* = .73, η_*p*_^2^ = .008 (Fig. S2).

## General Discussion

In the present research, we used single-pulse TMS to interfere with the causal chain from perception to action, finding an action-specific contribution to confidence in a visual discrimination task. Whereas previous work has focused on the contribution of sensory information to confidence, our results show that action-specific cortical representations also contribute to perceptual confidence.

Specifically, perturbation of premotor representations associated with responses in a visual discrimination task led to systematic changes in confidence despite discrimination accuracy remaining unchanged. Similar effects were seen when TMS pulses were applied both before and immediately after a visual discrimination, in accordance with the importance of postdecisional processes for determining confidence (Pleskac & Busemeyer, 2010). By including a no-TMS control condition in Experiment 2, we found that this change was predominantly driven by divergent effects of incongruent and congruent TMS on confidence for trials with correct responses. Specifically, TMS applied to the hemisphere incongruent with (ipsilateral to) the selected response reduced confidence, whereas TMS applied to the congruent (contralateral) hemisphere increased confidence. However, in both experiments, there was some evidence for an opposite pattern on trials with errors, such that the effects of TMS congruence interacted with decision accuracy. It is possible that less-consistent effects were observed on trials with errors because of reduced power to detect an effect on these trials, which by design were less numerous (~25%). We additionally found that metacognitive efficiency was reduced on incongruent trials independently of visual discrimination performance.

The mechanism by which action-specific TMS perturbs confidence remains to be determined. Dynamic models of the decision process suggest that confidence is based on the balance of evidence between competing accumulators supporting one or another choice option (Merkle & Van Zandt, 2006; Vickers, 1979). In this view, if TMS boosts or reduces the evidence for or against a choice, postresponse confidence would be altered in a systematic manner. For example, on incongruent trials, boosting the evidence supporting the unchosen response representation would tend to reduce confidence, whereas boosting the evidence in support of the chosen response on congruent trials may increase response confidence (Figs. 3a and 3b). However, this account cannot explain why effects of TMS were more pronounced on trials with correct responses compared with trials containing errors (Fig. 3c). An alternative possibility is that incongruence between TMS and the response hand leads to increased error or noise in confidence ratings (Erev, Wallsten, & Budescu, 1994), which could lead to a regression toward the mean on trials with both correct responses and errors and the observed decrease in metacognitive accuracy (Fig. 4).

More broadly, our data are consistent with the finding that the fluency of an action affects metacognitive assessments such as confidence or sense of control (Oppenheimer, 2008). For example, insertion of a subliminal arrow cue pointing in the opposite direction than the required motor response reduces participants’ sense of control, whereas an arrow in the same (congruent) direction increases sense of control (Wenke, Fleming, & Haggard, 2010). However, the manipulation of fluency in these studies was achieved via a perceptual cue, albeit subliminal, and therefore it is not possible to distinguish whether the cue’s effects on metacognitive judgments of control were due to effects on perception, action, or both. Here, by directly stimulating action-specific representations in premotor cortex during and after a visual discrimination, we demonstrated that direct perturbation of effector-specific representations alter metacognitive judgments of perceptual confidence.

Action-specific effects were observed at PMd but not at M1, which suggests that interfering with motor function per se may not affect metacognition. Instead, it appears that higher-level action representations in PMd (Graziano, Taylor, Moore, & Cooke, 2002; Passingham, 1993) may contribute to subjective confidence. PMd is implicated in the linking of stimuli to actions (Halsband & Passingham, 1985) and contains graded activity associated with the evolution of a decision (Cisek & Kalaska, 2005; Hernandez et al., 2002). It is also directly connected to the lateral prefrontal cortex, which has been linked to metacognition (Fleming et al., 2010, 2012; Rounis et al., 2010). In contrast, there is no direct connection between dorsolateral prefrontal cortex and M1 (Passingham, 1993), and M1 activity is more tightly linked to the motor response itself, which may explain why it did not contribute to confidence estimates in visual discrimination.

One potential limitation of our study is that brain-imaging-guided methods were not available to localize the target of stimulation. We can be confident that M1 stimulation is on target, because of the elicitation of specific twitches in the relevant muscles of the contralateral hand. Anterior to this location, PMd is the most plausible region to show hand-specific effects. We note that there are neurons associated with unilateral space in neighboring regions of the prefrontal cortex, such as the frontal eye field (Ro, Cheifet, Ingle, Shoup, & Rafal, 1999; Ro, Farnè, & Chang, 2002). However, the absence of performance differences between congruent and incongruent conditions, and the replication of the findings from Experiment 1 in a nonspatial version of the task in Experiment 2, makes a spatial-attentional explanation of our findings unlikely. Thus, we believe that the site of effective stimulation is PMd, in line with previous studies using similar methods of localization for this area (Bestmann et al., 2005; Chen et al., 2003; Johansen-Berg et al., 2002; O’Shea et al., 2007; Schluter et al., 1998).

Together, our findings reveal that action-specific decision signals contribute to visual confidence. The observation of action-specific contributions to confidence places constraints on future models of confidence and metacognition.

## Supplementary Material

Supplementary material

## Supplementary Material

Supplementary material
