# Supplementary material for: Action-Specific Disruption of Perceptual Confidence
Source: Psychol Sci. 2015 Jan;26(1):89–98. doi: 10.1177/0956797614557697 (PMC4361353; doi:10.1177/0956797614557697)
Supplement: Supplementary material [file DS_10.11770956797614557697_SupplementalMaterial.pdf]

DOI: 10.1177/0956797614557697

Action-specific disruption of perceptual confidence

Stephen M. Fleming, Brian Maniscalco, Yoshiaki Ko, Namema Amendi,
Tony Ro, Hakwan Lau

SUPPLEMENTAL ONLINE MATERIAL

DOI: 10.1177/0956797614557697

SUPPLEMENTARY METHODS

Meta-*d'* estimation

Meta-d' provides a response-bias free measure of how well confidence ratings track task accuracy (Maniscalco & Lau, 2012). In order to estimate meta-*d'* for each response separately (congruent, incongruent), we employed a response-specific meta-*d'* model (Maniscalco & Lau, 2014). Response-specific meta-*d'* is estimated by fitting the distribution of confidence ratings for each subject conditional on the discrimination response being correct or incorrect, separately for congruent and incongruent responses. A comprehensive overview of meta-*d'* and its response-specific variant is provided in Maniscalco & Lau (2014).

The fitting of meta-*d'* rests on calculating the likelihood of the confidence rating data given a particular "type 2" signal detection theoretic model. While conventional "type 1" SDT considers how well an observer can discriminate objective states of the world, such as stimulus present or absent, type 2 SDT characterises an observer's ability to discriminate her own correct or incorrect responses. Consider the simple case where the observer rates confidence as either "high" or "low." We can then distinguish 4 possible outcomes in the type 2 task: high confidence correct trials, low confidence correct trials, low confidence incorrect trials, and high confidence incorrect trials. By direct analogy with the type 1 analysis, we may refer to these outcomes as type 2 hits, type 2 misses, type 2 correct rejections, and type 2 false alarms, respectively.

Type 2 hit rate (HR) and type 2 false alarm rate (FAR) summarize an observer's type 2 performance and may be calculated as

$$type\ 2\ HR = HR_2 = p(high\ conf \mid stim = resp) = \frac{n(high\ conf\ correct)}{n(correct)}$$

$$type\ 2\ FAR = FAR_2 = p(high\ conf \mid stim \neq resp) = \frac{n(high\ conf\ incorrect)}{n(incorrect)}$$

DOI: 10.1177/0956797614557697

Confidence rating data may be richer than binary classification. In the general case, discrete confidence ratings may be provided on an ordinal scale from 1 to H, where 1 is the lowest confidence value and $H \geq 2$. (In the current experiment, H = 4.) In these cases the full type 2 ROC can be calculated by arbitrarily selecting a value h, $1 < h \leq H$, such that all confidence ratings greater than or equal to h are classified as "high confidence" and all others, "low confidence." Each choice of h generates a type 2 (FAR, HR) pair, and so calculating these for multiple values of h allows for the construction of a type 2 ROC curve with multiple points. A particular type 2 ROC can be generated by systematic variation of type 1 SDT parameters *d'* and *c*, and type 2 criteria c_2 (Galvin et al., 2003; Maniscalco & Lau, 2012). Describing the observed type 2 ROC in terms of these type 1 SDT parameters underpins the meta-*d'* model. By convention, the prefix "meta-" is added to each type 1 SDT parameter in order to indicate that the parameter is being used to fit type 2 ROC curves. Thus, the type 1 SDT parameters *d' c*, and c_2, when used to characterize type 2 ROC curves, are named meta-*d'* meta-*c*, and meta-c_2.

The equations below describe the calculation of type 2 probabilities from the type 1 SDT model for both S1 and S2 responses, e.g. congruent and incongruent in our experiment. For notational convenience, below we express these probabilities in terms of the standard SDT model parameters, omitting the "meta" prefix.

For a discrete confidence scale ranging from 1 to H, $H - 1$ type 2 criteria are required to rate confidence for each response type. Define type 2 confidence criteria for S1 and S2 responses as:

$$\dot{\underline{\boldsymbol{c}}}_{2,"S1"} = \left(c, c_{2,"S1"}^{conf=2}, c_{2,"S1"}^{conf=3}, \ldots, c_{2,"S1"}^{conf=H}, -\infty\right)$$

$$\dot{\underline{\boldsymbol{c}}}_{2,"S2"} = \left(c, c_{2,"S2"}^{conf=2}, c_{2,"S2"}^{conf=3}, \ldots, c_{2,"S2"}^{conf=H}, \infty\right)$$

and

$$\underline{\boldsymbol{c}}_{ascending} = \left(c_{2,"S1"}^{conf=H}, c_{2,"S1"}^{conf=H-1}, \ldots, c_{2,"S1"}^{conf=1}, c, c_{2,"S2"}^{conf=1}, c_{2,"S2"}^{conf=2}, \ldots, c_{2,"S2"}^{conf=H}\right)$$

DOI: 10.1177/0956797614557697

Then

$$Prob(conf = y \mid stim = S1, resp = "S1") = \frac{\Phi\left(\underline{\dot{\boldsymbol{c}}}_{2,"S1"}(y), -\frac{d'_{S1}}{2}\right) - \Phi\left(\underline{\dot{\boldsymbol{c}}}_{2,"S1"}(y+1), -\frac{d'_{S1}}{2}\right)}{\Phi\left(c, -\frac{d'_{S1}}{2}\right)}$$

$$Prob(conf = y \mid stim = S2, resp = "S1") = \frac{\Phi\left(\underline{\dot{\boldsymbol{c}}}_{2,"S1"}(y), \frac{d'_{S1}}{2}\right) - \Phi\left(\underline{\dot{\boldsymbol{c}}}_{2,"S1"}(y+1), \frac{d'_{S1}}{2}\right)}{\Phi\left(c, \frac{d'_{S1}}{2}\right)}$$

$$Prob(conf = y \mid stim = S1, resp = "S2") = \frac{\Phi\left(\underline{\dot{\boldsymbol{c}}}_{2,"S2"}(y+1), -\frac{d'_{S2}}{2}\right) - \Phi\left(\underline{\dot{\boldsymbol{c}}}_{2,"S2"}(y), -\frac{d'_{S2}}{2}\right)}{1 - \Phi\left(c, -\frac{d'_{S2}}{2}\right)}$$

$$Prob(conf = y \mid stim = S2, resp = "S2") = \frac{\Phi\left(\underline{\dot{\boldsymbol{c}}}_{2,"S2"}(y+1), \frac{d'_{S2}}{2}\right) - \Phi\left(\underline{\dot{\boldsymbol{c}}}_{2,"S2"}(y), \frac{d'_{S2}}{2}\right)}{1 - \Phi\left(c, \frac{d'_{S2}}{2}\right)}$$

Next we consider the procedure for finding the parameters of the type 1 SDT model that maximize the likelihood of the response-specific type 2 data for a particular response, "S1" (e.g. congruent in our experiments). The same procedure can be applied to estimate meta-*d'* for "S2" (e.g. incongruent) responses. The likelihood of the type 2 confidence data can be characterized using the multinomial model as

DOI: 10.1177/0956797614557697

$$L_{"S1"}(\theta_{"S1"}|data) \propto \prod_{y,s} Prob_{\theta}(conf = y \mid stim = s, resp = "S1")^{n_{data}(conf=y|stim=s,resp="S1")}$$

Maximizing likelihood is equivalent to maximizing log-likelihood, and in practice it is typically more convenient to work with log-likelihoods. The log-likelihood for type 2 data is given by

$$\log L_{"S1"}(\theta_{"S1"}|data) \propto \sum_{y,s} n_{data} \log Prob_{\theta}$$

$\theta_{"S1"}$ is the set of parameters for the response-specific meta-SDT model:

$$\theta_{"S1"} = (\text{meta}d'_{"S1"}, \text{meta}c_{"S1"}, \text{meta}\underline{\boldsymbol{c}}_{2,"S1"})$$

$n_{data}(conf = y \mid stim = s, resp = "S1")$ is a count of the number of times in the data a confidence rating of y was provided when the stimulus was s and response was “S1”. y and s are indices ranging over all possible confidence ratings and stimulus classes, respectively.

The preceding approach for quantifying type 2 sensitivity with the type 1 SDT model—i.e. for fitting the meta-SDT model—can then be summarized as an optimization problem:

$$\theta^*_{"S1"} = \underset{\theta_{"S1"}}{\arg\max}\ L_{2,"S1"}(\theta_{"S1"}|data),$$
$$\text{subject to:} \quad \text{meta}c'_{"S1"} = c', \quad \gamma(\text{meta}\underline{\boldsymbol{c}}_{ascending})$$

where $\text{meta}d'_{"S1"} \in \theta^*_{"S1"}$ measures type 2 sensitivity for “S1” responses, $\gamma(\text{meta}\underline{\boldsymbol{c}}_{ascending})$ is a Boolean function which returns a value of “true” only if the type

1 and type 2 criteria stand in appropriate ordinal relationships, i.e. each element in $\underline{\boldsymbol{c}}_{ascending}$ is at least as large as the previous element, and c' is a relative measure of type 1 response bias, *c' = c / d'*.

Matlab code for implementing this maximum likelihood procedure for fitting the response-specific meta-SDT model can be found at http://www.columbia.edu/~bsm2105/type2sdt.

References

Galvin, S. J., Podd, J. V., Drga, V., & Whitmore, J. (2003). Type 2 tasks in the theory of signal detectability: discrimination between correct and incorrect decisions. *Psychonomic Bulletin & Review*, *10*(4), 843–876.

Maniscalco, B., & Lau, H. (2012). A signal detection theoretic approach for estimating metacognitive sensitivity from confidence ratings. *Consciousness and Cognition*, *21*(1):422-430.

Maniscalco, B., & Lau, H. (2014). "Signal Detection Theory Analysis of Type 1 and Type 2 Data: Meta-d', Response-Specific Meta-d', and the Unequal Variance SDT Model," in *The Cognitive Neuroscience of Metacognition*, eds. S. M. Fleming and C. D. Frith (Springer).

DOI: 10.1177/0956797614557697

FIGURES

Figure S1

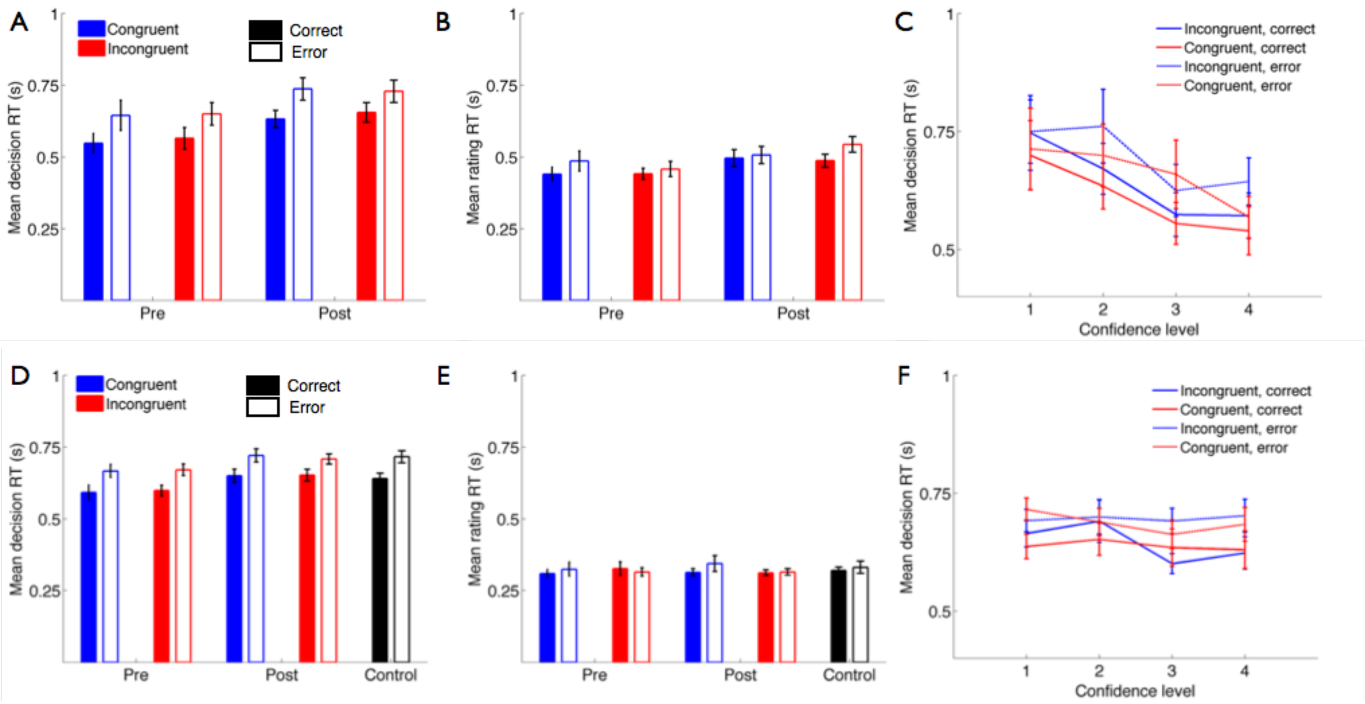

Experiment 1 (A-C) and 2 (D-F) PMd group response times by condition. (A, D) Response times for the visual discrimination sorted by TMS condition and accuracy. (B, E) Response times for the confidence rating sorted by TMS condition and accuracy of the preceding visual discrimination response. (C, F) Visual discrimination response times plotted as a function of confidence level. Error bars reflect standard errors of the mean.

DOI: 10.1177/0956797614557697

Figure S2

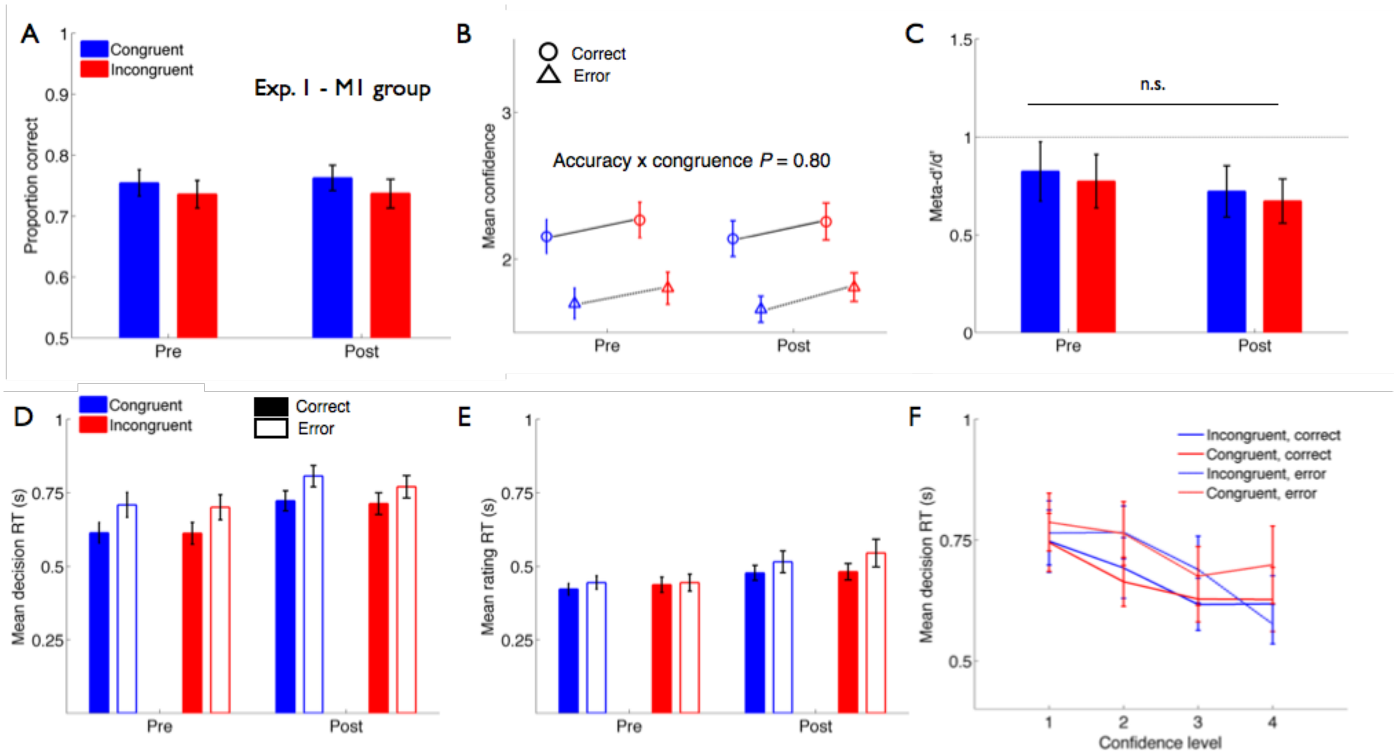

Experiment 1 M1 group data. (A) Proportion correct sorted by condition. (B) Mean confidence sorted by TMS condition and response accuracy. (C) Metacognitive sensitivity (meta-*d'*/*d'*) calculated separately for congruent and incongruent premotor cortex TMS trials in the pre- and post-decision stimulation conditions. (D) Response times for the visual discrimination sorted by TMS condition and accuracy. (E) Response times for the confidence rating sorted by TMS condition and accuracy of the preceding visual discrimination response. (F) Visual discrimination response times plotted as a function of confidence level. Error bars reflect standard errors of the mean.

DOI: 10.1177/0956797614557697

Figure S3

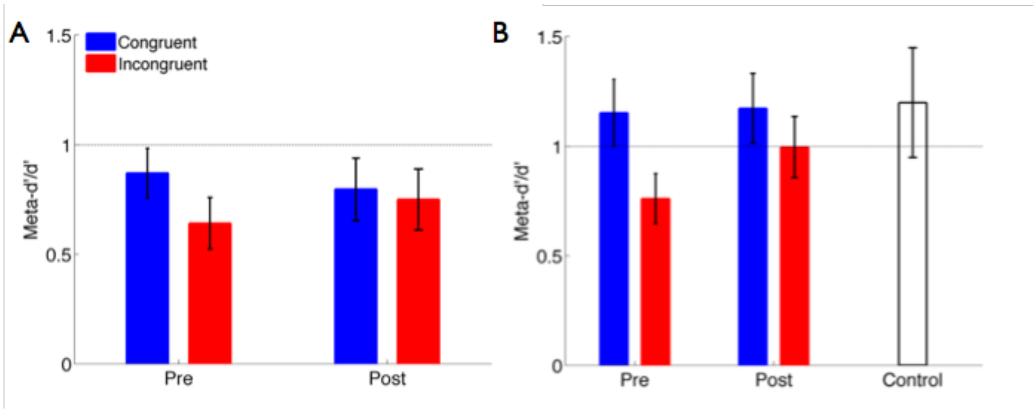

Metacognitive sensitivity (meta-*d'*/*d'*) plotted separately for the PMd group of Experiment 1 (A) and Experiment 2 (B). Error bars reflect standard errors of the mean.

DOI: 10.1177/0956797614557697

TABLES

Table S1

Experiment	*TMS time*	*TMS congruence*	*Accuracy*	*Mean (SD) confidence*
Experiment 1 - PMd	Pre-decision	Congruent	Correct	2.63 (0.46)
			Incorrect	1.93 (0.46)
		Incongruent	Correct	2.59 (0.52)
			Incorrect	2.11 (0.58)
	Post-decision	Congruent	Correct	2.60 (0.52)
			Incorrect	1.88 (0.45)
		Incongruent	Correct	2.56 (0.53)
			Incorrect	1.97 (0.54)
Experiment 1 – M1	Pre-decision	Congruent	Correct	2.16 (0.48)
			Incorrect	1.70 (0.42)
		Incongruent	Correct	2.27 (0.50)
			Incorrect	1.80 (0.45)
	Post-decision	Congruent	Correct	2.14 (0.50)
			Incorrect	1.66 (0.36)
		Incongruent	Correct	2.26 (0.52)
			Incorrect	1.81 (0.40)
Experiment 2 – PMd	Pre-decision	Congruent	Correct	2.88 (0.50)
			Incorrect	1.88 (0.34)
		Incongruent	Correct	2.68 (0.46)
			Incorrect	2.03 (0.45)
	Post-decision	Congruent	Correct	2.99 (0.38)
			Incorrect	1.93 (0.41)
		Incongruent	Correct	2.78 (0.50)
			Incorrect	1.88 (0.43)
	Control	Control	Correct	2.85 (0.42)
			Incorrect	1.93 (0.45)

Summary of confidence by experiment and condition. SD indicates standard deviation of means across subjects.

DOI: 10.1177/0956797614557697

Table S2

Experiment	*TMS time*	*TMS congruence*	*Mean (SD) meta-d'/d'*
Experiment 1 - PMd	Pre-decision	Congruent	0.87 (0.47)
		Incongruent	0.64 (0.49)
	Post-decision	Congruent	0.80 (0.59)
		Incongruent	0.75 (0.57)
Experiment 1 – M1	Pre-decision	Congruent	0.82 (0.62)
		Incongruent	0.77 (0.56)
	Post-decision	Congruent	0.72 (0.54)
		Incongruent	0.67 (0.47)
Experiment 2 – PMd	Pre-decision	Congruent	1.15 (0.65)
		Incongruent	0.76 (0.49)
	Post-decision	Congruent	1.17 (0.67)
		Incongruent	1.00 (0.59)
	Control	Control	1.20 (1.06)

Summary of metacognitive efficiency (meta-*d'/d'*) by experiment and condition.

Table S3

Experiment	*Confidence rating*	*Mean (SD)*
Experiment 1 - PMd	1	88.4 (59.5)
	2	96.6 (45.6)
	3	105.3 (52.5)
	4	80.8 (64.6)
Experiment 1 – M1	1	135.2 (78.7)
	2	94.9 (37.0)
	3	86.7 (48.8)
	4	43.1 (46.6)
Experiment 2 – PMd	1	116.2 (42.7)
	2	88.4 (34.1)
	3	110.2 (54.2)
	4	157.2 (67.0)

Means and SDs of rating counts for each confidence level separately for each experiment.

DOI: 10.1177/0956797614557697

Tables S4

Regression coefficients for the influence of *accuracy*, *congruence* and *time* on visual discrimination response times in each experimental group. **, $P < 0.01$; *, $P < 0.05$.

PMd group, Experiment 1

Coefficient	*Estimate (SE)*	*P-value*
(Intercept)	0.66 (0.041)	< 0.0001**
accuracy	-0.091 (0.024)	< 0.001**
congruence	-0.008 (0.029)	0.77
time	0.073 (0.023)	0.0013**
accuracy × congruence	-0.010 (0.034)	0.78
accuracy × time	0.017 (0.021)	0.41
congruence × time	0.003 (0.039)	0.94
accuracy × congruence × time	-0.008 (0.039)	0.83

M1 group, Experiment 1

Coefficient	*Estimate (SE)*	*P-value*
(Intercept)	0.69 (0.043)	< 0.0001**
accuracy	-0.081 (0.022)	< 0.001**
congruence	0.004 (0.025)	0.89
time	0.072 (0.022)	0.0014**
accuracy × congruence	-0.003 (0.022)	0.87
accuracy × time	0.026 (0.022)	0.23
congruence × time	0.030 (0.022)	0.18
accuracy × congruence × time	-0.018 (0.027)	0.52

PMd group, Experiment 2

Coefficient	*Estimate (SE)*	*P-value*
(Intercept)	0.67 (0.020)	< 0.0001**
accuracy	-0.075 (0.015)	< 0.0001**
congruence	0.004 (0.024)	0.87
time	0.037 (0.026)	0.14
accuracy × congruence	-0.010 (0.024)	0.67
accuracy × time	0.016 (0.017)	0.33
congruence × time	0.020 (0.023)	0.40
accuracy × congruence × time	-0.016 (0.026)	0.55

Tables S5

Regression coefficients for the influence of *accuracy*, *congruence* and *time* on confidence rating response times in each experimental group. **, $P < 0.01$; *, $P < 0.05$.

PMd group, Experiment 1

Coefficient	*Estimate (SE)*	*P-value*
(Intercept)	0.46 (0.025)	< 0.0001**
accuracy	-0.023 (0.019)	0.21
congruence	0.026 (0.028)	0.35
time	0.081 (0.024)	< 0.001**
accuracy × congruence	-0.022 (0.027)	0.42
accuracy × time	-0.034 (0.026)	0.19
congruence × time	-0.064 (0.033)	0.050*
accuracy × congruence × time	0.068 (0.036)	0.062

M1 group, Experiment 1

Coefficient	*Estimate (SE)*	*P-value*
(Intercept)	0.45 (0.027)	< 0.0001**
accuracy	-0.017 (0.016)	0.29
congruence	-0.012 (0.022)	0.57
time	0.086 (0.028)	0.0023**
accuracy × congruence	-0.005 (0.024)	0.83
accuracy × time	-0.044 (0.030)	0.14
congruence × time	-0.016 (0.031)	0.61
accuracy × congruence × time	0.030 (0.036)	0.41

PMd group, Experiment 2

Coefficient	*Estimate (SE)*	*P-value*
(Intercept)	0.32 (0.016)	< 0.0001**
accuracy	0.008 (0.015)	0.57
congruence	0.003 (0.016)	0.85
time	-0.003 (0.014)	0.81
accuracy × congruence	-0.018 (0.025)	0.47
accuracy × time	-0.010 (0.020)	0.59
congruence × time	0.024 (0.019)	0.22
accuracy × congruence × time	-0.007 (0.022)	0.75
